# Editorial: Fertilization in the spotlight: Dynamics and mechanisms of sperm-egg interaction

**DOI:** 10.3389/fcell.2022.993865

**Published:** 2022-09-08

**Authors:** Enrica Bianchi, Maria Jimenez-Movilla, Amber R. Krauchunas

**Affiliations:** ^1^ Department of Biology, Hull York Medical School, York Biomedical Research Institute, University of York, York, United Kingdom; ^2^ Department of Cell Biology and Histology, Faculty of Medicine, University of Murcia, IMIB, Murcia, Spain; ^3^ Department of Biological Sciences, University of Delaware, Newark, DE, United States

**Keywords:** fertilization, sperm, egg, gamete interaction, cell fusion, acrosome

Fertilization has intrigued scientists for centuries and is of great interest to the general public for its wider implications in human health. Undeniably, outstanding progress has been made but many questions remain to be answered. The purpose of this Research Topic was to highlight the variety of molecular mechanisms underlying gamete interaction in sexually reproducing species and to draw attention to the long-standing open questions of fertilization.

Unicellular and multicellular species use fertilization to ensure the formation of a unique new organism that is genetically distinct from their parents. Two purposefully developed cells collectively named gametes (sperm and eggs in multicellular organisms) meet either in the external environment or in internal organs, bind to one another and fuse together to generate a viable progeny.

Thanks to decades of research we now have the ability to manipulate gametes *in vitro* and *in vivo* and to successfully achieve *in vitro* fertilization in humans as well as in many other species (Carroll, 2018). Our knowledge of the molecular mechanisms governing the sperm-egg interaction has greatly increased particularly with the advent of genetically modified animal models which contributed to identify molecules that are essential for fertilization. Among the breakthroughs of the last decade are the discovery of the first binding pair essential for mammalian fertilization (Juno and Izumo1) (Bianchi et al., 2014), and the identification of the first fusogen (a protein that induces cell fusion) named Hap2/GCS1 in plants and unicellular organisms (Johnson et al., 2004; Mori et al., 2006; von Besser et al., 2006; Liu et al., 2008). Investigations in organisms as different as C. elegans, Zebrafish, Abalone, Sea urchin (Krauchunas et al., 2016; Raj et al., 2017; Deneke and Pauli, 2021; Wessel et al., 2021) and mammals (Bianchi and Wright, 2020) are contributing to identifying novel proteins involved in sperm-egg recognition and to unravel the dynamics of these interactions.

Even today, knowledge about the molecular mechanisms that produce the capacity of recognition, union and fusion between the two cells, the oocyte and the spermatozoa, is still very scarce and any solid contribution can help to unravel the molecular puzzle that is the origin of the zygote. On the side of spermatozoa, the research conducted by Wang et al. identified Chromosome 1 open reading frame 56 (C1orf56) to be a SLeX-binding sperm protein. They showed that purified C1orf56 from spermatozoa bound to human zona pellucida (ZP) and immunofluorescence staining localized C1orf56 to the acrosomal region of capacitated spermatozoa, a region that binds ZP. Moreover, they found that C1orf56 is relocated from the equatorial region to the acrosomal region after capacitation suggesting that C1orf56 may have functions after ZP-binding and acrosome reaction.

Two contributions unravel the role of IZUMO1 and SPACA6 using mutant sperm lacking these fusion-related genes previously demonstrated in a mouse model. The research conducted by Matsumara et al. show here that Izumo1 KO male rats are infertile due to fertilization defects. However, unlike in mice, Izumo1 knockout rat spermatozoa failed to bind to the oolemma. Moreover, reanalysis of the Izumo1 KO mice shows the percentage of the oolemma bound acrosome-reacted spermatozoa was drastically decreased in the Izumo1 KO mice compared with WT, indeed suggesting that IZUMO1 is required for binding the acrosome-reacted spermatozoa to oolemma prior to fusion. Of note, it was reported that the acrosome-reacted spermatozoa hardly bind to the JUNO KO eggs in mice (Bianchi et al., 2014). Intriguingly, this study found that, unlike sperm lacking Izumo1, sperm lacking the novel fusion-related genes Fimp, Sof1, Spaca6, or Tmem95 bind to the oolemma after the acrosome reaction. All together these data suggest that these proteins could be involved in different molecular pathways to regulate binding and/or fusion since all of them are essential to complete fertilization. The original article published by Binner et al. using the Zebrafish model shows that Spaca6 knockout males are sterile. While sperm lacking Spaca6 have normal morphology and are motile, Spaca6-deficient sperm fail to bind to the egg and therefore cannot complete fertilization. This is in contrast to murine sperm lacking SPACA6, which was reported to be able to bind but unable to fuse with oocytes (Barbaux et al., 2020; Noda et al., 2020). Moreover, here they show that Spaca6 regulates Dcst2 protein levels and interestingly, recent work in mice has shown that SPACA6 levels are decreased in sperm lacking IZUMO1, DCST1 and/or DCST2 (Inoue et al., 2021). Then authors suggest that Spaca6 may contribute to forming and/or stabilizing a multi-factor complex on the sperm membrane that regulates both binding and fusion.

The study conducted by Gonzalez-Brusi et al. identified by mass spectrometry a list of 41 sperm proteins that were pulled down with TMEM95 and none of them were sperm proteins known to play a role in fertilization, suggesting an independent role of TMEM95 in fertilization. Between these lists, they propose OLFR601 protein as a candidate to collaborate with TMEM95, as OLFR601 is allocated to the acrosomal region and may mediate affinity for an odorant involved in fertilization. However, Olfr601 disruption did not impair the sperm fertilization ability, suggesting that its function may be redundant with that of other sperm proteins. Nevertheless, more studies are needed to further investigate the complex functions of those newly identified fusion-related molecules.

Regarding the oocyte side, in silico docking analysis by Stepanenko et al. for blocking JUNO-IZUMO1 interaction identifies two molecules, Z786028994 and Z1290281203, that show fertilization inhibitory effect in both an *in vitro* fertilization assay in mice and an *in vitro* penetration of human sperm into hamster oocytes. The accumulation of sperm cells in the perivitelline space of eggs treated with molecules Z786028994 and Z1290281203 suggests that the fertilization failure seen by these two molecules is a result of inhibition of sperm–egg fusion. However, none of the molecules significantly affected the binding of JUNO and IZUMO1 using AVEXIS. Therefore, until further research is performed, the mechanism of action of these IVF inhibitors remains unclear.

In this Research Topic, three research articles focus on cellular characteristics of sperm that contribute to the sperm’s fertilizing ability. Giaccagli et al. examined the relationship between mitochondrial activity and fertilizing ability of the sperm. Their results indicate that there is a rise in mitochondrial membrane potential during sperm capacitation and this mitochondrial activity is important for both *in vitro* and *in vivo* fertilization at the step of zona pellucida penetration. Ma et al. tested the role of Toll-like receptor 2 (TLR2) for sperm function and similarly concluded that TLR2 plays a role in sperm interactions with the zona pellucida and suggest that TLR2 contributes to acrosomal exocytosis in response to zona pellucida attachment. Structural analysis of acrosomal exocytosis was carried out by Leung et al. with the use of cryoelectron tomography to visualize acrosomal exocytosis in pig sperm. In addition to observing a heterogenous population of vesicles and paracrystalline patches surrounding the fully acrosome-reacted sperm, this study shows that the post-acrosomal plasma membrane becomes densely packed with membrane protein densities that were not present in unreacted cells.

To fully understand sperm-egg interactions we need to not only investigate the molecular and cellular aspects of the gametes, but to examine the evolution of reproductive genes. Two original research articles and one review in this Research Topic highlight the importance of gene duplication and diversification of sperm-expressed genes in different taxa. Carlisle et al. carried out a detailed evolutionary genomic analysis in abalone and discovered duplications of lysin and sp18 ancestral to abalone. Interestingly, they did not find evidence of recent duplications of egg coat proteins suggesting that it is not duplications on the egg side that are driving duplication and diversification of the sperm acrosomal proteins in *H. tuberculata*. Transcriptomic and proteomic analyses were carried out by Wilburn et al. to provide a molecular description of salamander gametes. Their data reveal that the sperm express paralogs of pheromone proteins suggesting these protein families have been co-opted for multiple reproductive functions through gene duplication and rapid evolution. In addition, gene duplication and repeat domain expansion in the evolution of fertilization proteins such as Izumo, Juno, DCST, and ZP domains is reviewed by Rivera and Swanson.

Four additional reviews round out this spotlight on sperm-egg interactions. Brukman et al. review the fusexin class of fusogens within the context of structure, mechanism of action, and gamete fusion. Pinello and Clark review gamete fusion and the role of HAP2/GCS1 in *Chlamydomonas reinhardtii* and *Tetrahymena thermophila* as well as discuss the possibility of HAP2/GCS1 as a candidate for transmission-blocking vaccine development against parasitic protists. Gonzalez et al. review the role of CRISP proteins in fertility and different stages of the fertilization process discussing both genetic and non-genetic studies to dissect the functions of this protein family. Finally, Saito and Sawada review sperm-egg interactions and self/nonself-recognition at the level of the egg coat in ascidians.

The broad range of subjects in this research topic show the great effort of the scientific community in elucidating the still elusive mechanisms of fertilization. We believe that the collaboration of researchers working on various aspects of sperm-egg interaction is instrumental to unravel the molecular cascades orchestrating this event in different species and to overcome the experimental limitations of investigating gamete biology.
